# Renal Cell Carcinoma With Fibromyomatous Stroma: A New Case

**DOI:** 10.7759/cureus.32238

**Published:** 2022-12-05

**Authors:** Mohamed Amine Haouane, Fouad Hajji, Omar Ghoundale, Mohamed Amine Azami

**Affiliations:** 1 Department of Pathology, Cadi Ayyad University/Avicenne Military Hospital, Marrakech, MAR; 2 Department of Urology, Cadi Ayyad University/Ibn Sina Military Hospital, Marrakech, MAR; 3 Department of Urology, Cadi Ayyad University/Avicenne Military Hospital, Marrakech, MAR; 4 Department of Pathology, Cadi Ayyad University/Ibn Sina Military Hospital, Marrakech, MAR

**Keywords:** surgery, cancer, fibromyomatous stroma, renal cell carcinoma, kidney

## Abstract

In the 2016 World Health Organization (WHO) classification of renal tumors, renal cell carcinoma with fibromyomatous stroma (RCC FMS) (formerly RCC with leiomyomatous or smooth muscle stroma) was classified as an emerging or provisional entity of renal cell carcinoma (RCC).

We report a rare case of RCC FMS in a 62-year-old male patient with hypertension, type II diabetes mellitus, and early chronic kidney disease. He was referred to the Department of Urology for an incidental finding of a 2-cm-long left renal nodule on a routine abdominal ultrasound. A laparoscopic right partial nephrectomy was performed. Histopathology and immunohistochemistry studies confirm the diagnosis of RCC FMS.

The purpose of this work is to review and discuss newly acquired data and evidence on the clinical, pathological, immunohistochemical, molecular, and prognostic aspects of this unusual entity in the hopes of assisting pathologists in accurate diagnosis.

## Introduction

Renal cell carcinoma (RCC) accounts for about 3% of all adult cancers and 90%-95% of kidney neoplasms [1]. In the 2016 World Health Organization (WHO) classification of renal tumors, the RCC subtype was largely updated based on morphological and molecular criteria. Renal cell carcinoma with fibromyomatous stroma (RCC FMS) was classified here as a provisional entity [2].

Based on the existing literature, it is an exceptionally rare histopathological entity that was initially identified by Canzonieri et al. in 1993 [3], and the first case series of five cases was reported by Kuhn et al. in 2006 [4].

This paper hopes to further facilitate the identification of this entity in practice so that pathologists will be familiar with this emerging entity before it becomes a confirmed entity by the next WHO classification of renal neoplasm.

## Case presentation

A 62-year-old Moroccan male patient with hypertension, type II diabetes mellitus, and early chronic kidney disease was referred for urological evaluation for an incidental finding of a 2-cm-long left renal nodule on a routine abdominal ultrasound. Laboratory testing showed renal impairment with a serum creatinine of 90 µmol/L and an estimated glomerular filtration rate (eGFR) of 95 mL/minute/1.73 m^2^. The patient had no symptoms. He did not have any documented family history of cancer or kidney tumors.

**Figure 1:**
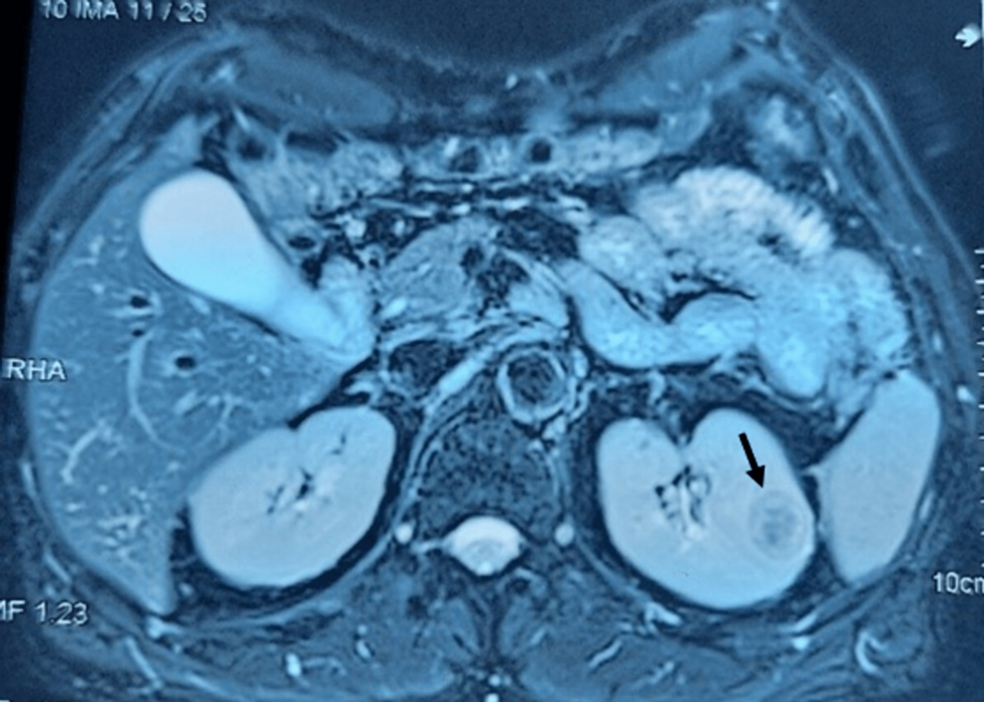
Abdominal MRI showing a mid-cortical nodular lesion measuring 23 mm (black arrow) discreetly enhanced after injection of contrast product of the left kidney. MRI: magnetic resonance imaging

Magnetic resonance imaging (MRI) of the left kidney revealed a mid-cortical nodular lesion measuring 23 mm, discreetly enhanced after injection of contrast product (Figure 1). The right kidney was normal; no lymphadenopathy, renal vein tumor thrombus, or metastatic lesions were detected.

A kidney biopsy under ultrasound guidance was performed to evaluate the tumor. The histopathological examination of the biopsy revealed an atypical epithelial tumor proliferation made of tubes and nests consisting of atypical tumor cells with clear cytoplasm. This microscopic aspect was in favor of renal cell carcinoma (RCC).

Due to his chronic renal disease and the small, cortical, and well-limited nature of the nodule, a laparoscopic partial left nephrectomy was considered safe from surgical complications.

**Figure 2:**
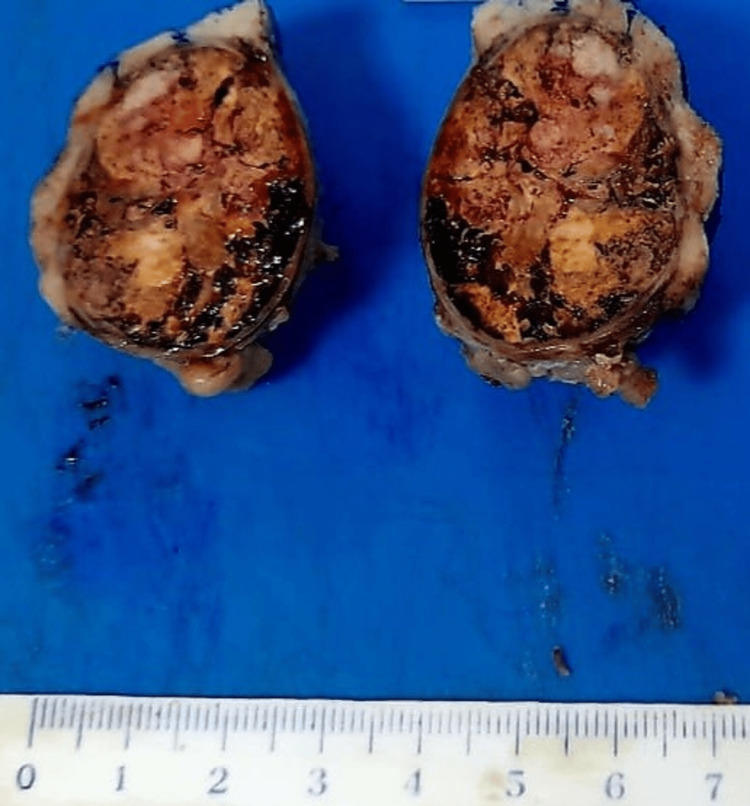
Gross pathological specimen showing a well-defined, encapsulated, brownish-white solid mass with some hemorrhagic foci surrounded by a border of the renal parenchyma.

We received a partial nephrectomy specimen weighing 10 g and measuring 28 mm × 25 mm, showing a well-defined tumor nodule measuring 25 mm × 24 mm, brownish in appearance with hemorrhagic areas and a renal surgical margin of 0.1 cm (Figure 2). The renal parenchymal margin was negative without tumor infiltration.

**Figure 3:**
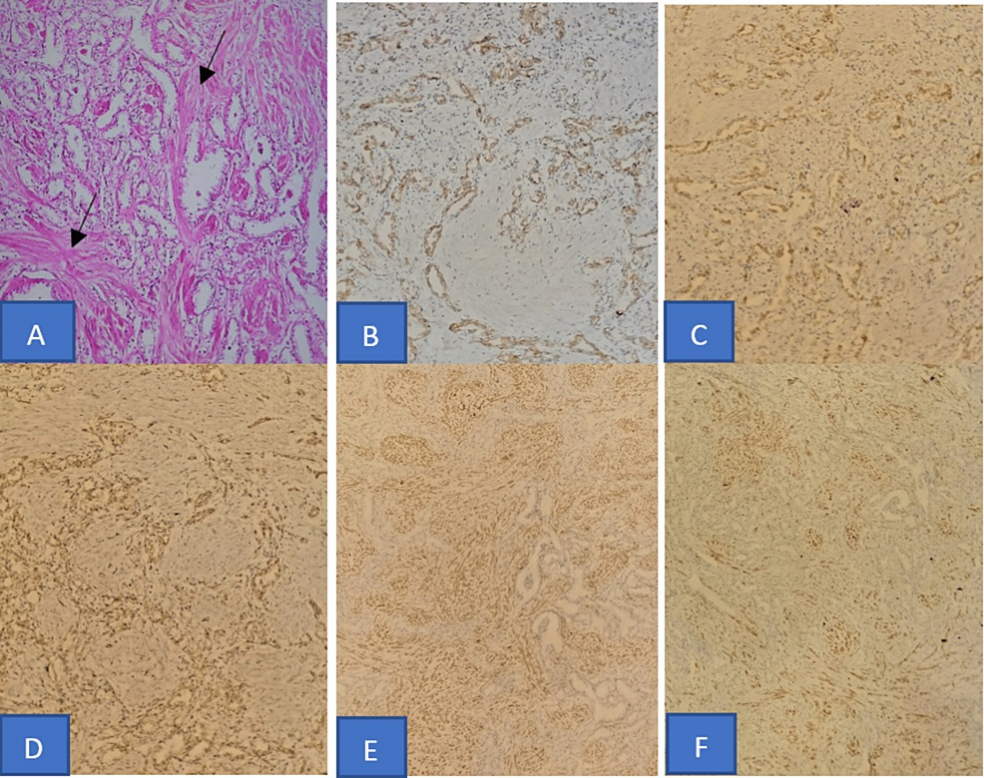
Histopathological aspects and immunohistochemical profile of renal cell carcinoma with fibromyomatous stroma. (A) The tumor is composed of abundant fibro-muscular stroma arranged in bundles (black arrow), intermixed with the epithelial component arranged in branching tubules, nests, and glands, lined by cells with clear cytoplasm and mild nuclear atypia (hematoxylin and eosin, 200×). Tumor epithelial cells are positive for (B) pan-cytokeratin, (C) cytokeratin 7, and (D) vimentin. The stroma cells showed diffuse staining with antibodies to (E) smooth muscle actin and (F) caldesmon.

The tumor is microscopically composed of abundant fibro-muscular stroma arranged in fascicles of spindle cells without atypia or mitosis, intermixed with the epithelial component, which was composed of branching tubules, nests, and glands lined by cells with clear cytoplasm and mild nuclear atypia (Fuhrman grade 2). There was no evidence of necrosis or neoplastic vascular emboli. The adjacent renal parenchyma appeared normal (Figure 3A).

An immunohistochemical study revealed that neoplastic epithelial cells showed strong and diffuse positivity for pan-cytokeratin (AE1/AE3), cytokeratin 7 (CK7), and vimentin (Figures 3B-3D). The staining for renal cell carcinoma (RCC) and cluster of differentiation 10 (CD10) were negative. The stromal cells were positive for smooth muscle actin and caldesmon (Figures 3E, 3F).

Based on these microscopic and immunohistochemical findings, the diagnosis of renal cell carcinoma with fibromyomatous stroma has been established, and the tumor was staged as pT1aNxMx (tumor, node, and metastasis (TNM) classification eighth edition). The patient has been under surveillance without recurrences or complications to date.

## Discussion

Renal cell carcinoma with fibromyomatous stroma (RCC FMS) was categorized as an “emerging/provisional renal entity,” “an RCC with (angio)leiomyomatous stroma,” in the 2016 WHO classification [5].

The various terminology and names used to characterize RCC with abundant smooth muscle stroma added to the complexity of this entity. Examples include mixed renal tumor with carcinomatous and fibro-leiomyomatous components, RCC associated with prominent angioleiomyoma-like proliferation, clear cell RCC with smooth muscle stroma, RCC with clear cells, smooth muscle stroma and negativity for 3p deletion, RCC with leiomyomatous stroma (RCCLS), and RCC with angio-leiomyomatous-like stroma [6].

There has been some disagreement in the past over whether RCC FMS is a separate entity or a group of RCCs with similar morphologies [7], but current findings have shown that RCC FMS is a distinct entity separate from other RCCs [8,9]. The epidemiological profile of RCC FMS has not been adequately investigated due to its uncommon occurrence. Nevertheless, according to the data, RCC FMS mainly affects people aged between 31 and 79, with a slight male predominance [10]. Macroscopically, in contrast to the polymorphic brown to golden yellow areas with hemorrhagic or necrosis observed in clear cell RCC, the cut surface of RCCLS displays solid white tissue with a “leiomyoma-like” appearance [11]. Histologically, RCC FMS is composed of two components that are often admixed: a stromal fibro-muscular component and an epithelial component [6].

The stromal component is variable, ranging from minimal to abundant, and is typically leiomyomatous or fibro-leiomyomatous. This stroma is non-neoplastic and reactive. The clonality of this stroma was also analyzed, and it was discovered to be polyclonal [12].

The epithelial component of this tumor comprises tumor nodules, which constitute elongated and frequently branching tubules and papillary structures lined with transparent or moderately eosinophilic cells with abundant cytoplasm. Cellular nuclei are usually grade 2, according to the WHO/International Society of Urological Pathology (ISUP) histological grading system for RCC [13].

Pathologists may find it difficult to make an exact morphological diagnosis of this entity, especially during small kidney biopsies. This distinction is crucial for the therapeutic decision (i.e., surveilling nephrectomy versus radical nephrectomy).

This entity is difficult to distinguish from a variety of benign and malignant neoplasms, such as clear cell renal cell carcinoma with degenerative fibrosis or hemangioma-like changes, sarcomatoid renal cell carcinoma, clear cell (tubulo)papillary RCC, renal angiomyoadenomatous tumors (RAT), epithelioid angiomyolipoma, and mixed epithelial stromal tumor of the kidney [14]. The distinction between these entities often requires an immunohistochemical study, the profile of which has varied greatly in the different cases published until today.

Immunohistochemically, the epithelial component is positive for pan-cytokeratin, CK7, epithelial membrane antigen (EMA), CD10, paired-box gene 8 (PAX8), and RCC. Similarly, smooth muscle actin, HHF35, and desmin are all positive in the stromal component [9].

Recently, several studies have analyzed RCC FMS at the molecular level. According to the recent study by Shah et al., in which 18 cases of RCC FMS were examined at the molecular level, these tumors can be divided into two molecular subgroups: a subgroup characterized by somatic mutations of tuberous sclerosis complex 1 (TSC1), TSC2, or mammalian target of rapamycin (MTOR) involving the TSC/MTOR pathway and a minority subgroup displaying Elongin C (ELOC) (transcription elongation factor B (TCEB1)) mutations frequently associated with monosomy for chromosome 8 [8,15-17]. Molecular genetic studies of RCC FMS reveal no von Hippel-Lindau (VHL) mutation, trisomy 7, or trisomy 17 in both molecular subgroups [18]. However, these tumors are often sporadic and rarely hereditary, occurring in patients with tuberous sclerosis complex (TSC) [8].

Although data is limited, clinical behavior and prognosis suggest the tumors to be generally non-aggressive, given the small-sized lesions and relatively lower tumor grades published to date.

## Conclusions

In summary, renal cell carcinoma with fibromyomatous stroma is a distinct and rare neoplasm with characteristic morphological, immunohistochemical, and molecular features and is included as a provisional entity in the 2016 World Health Organization (WHO) classification of renal epithelial neoplasia. However, further research and validation criteria are required before it can be defined as a confirmed entity in the next classification of renal tumors by the WHO.
